# An Imitation Too Perfect: Isolated Neurosarcoidosis Mimicking Spheno-Orbital Meningioma With Hyperostosis

**DOI:** 10.7759/cureus.93591

**Published:** 2025-09-30

**Authors:** Patrice Finet, Lina Daoud, Paul Desclée

**Affiliations:** 1 Neurosurgery, UCLouvain, Brussels, BEL; 2 Pathology and Laboratory Medicine, UCLouvain, Brussels, BEL; 3 Radiology, UCLouvain, Brussels, BEL

**Keywords:** extra-axial tumor, hyperostosis, neurosarcoidosis, sarcoidosis-like, spheno-orbital meningioma

## Abstract

We report a rare case of isolated neurosarcoidosis presenting as a spheno-orbital mass with hyperostosis of the sphenoid wing, initially misdiagnosed as a meningioma. Neurosarcoidosis may manifest as dural-based lesions that mimic tumors radiologically; however, hyperostosis, a hallmark of meningiomas, is usually absent. This unusual presentation causes diagnostic confusion. This case highlights the diagnostic challenges when sarcoidosis mimics meningiomas, particularly in the presence of hyperostosis. Awareness of such atypical presentations is crucial for accurate diagnosis and appropriate management.

## Introduction

Sarcoidosis is a multisystem, immune-mediated granulomatous disease of unknown etiology that affects the nervous system in approximately 5-10% of cases [1].

The clinical manifestations of central nervous system sarcoidosis, also referred to as neurosarcoidosis, vary depending on the anatomical location of the granulomatous lesions. These manifestations include involvement of the meninges, peripheral nerves (manifesting as clinical neuropathies), and the brain parenchyma, with lesions in the brain or spinal cord occurring without obvious meningeal involvement. In rare instances, dural inflammation (pachymeningitis) may present as a dural-based mass, clinically and radiologically mimicking a meningioma [2-8]. These lesions with isolated focal dural involvement often present diagnostic challenges on imaging studies [5].

Magnetic resonance imaging (MRI) plays a crucial role in identifying meningeal involvement, along with periventricular and intra- or extra-axial white matter lesions. Nevertheless, the wide range of neuroradiological presentations combined with nonspecific clinical symptoms often results in intracranial sarcoid granulomatosis being mistaken for primary brain tumors, such as gliomas, meningiomas, or intrasellar masses, or infectious diseases of the central nervous system [2,6,9].

Furthermore, skeletal involvement in sarcoidosis is relatively uncommon, and its prevalence is not well established. Osseous sarcoidosis can affect any part of the skeleton, though its distribution is variable. The lesions most commonly present as osteolytic, though sclerotic changes may also be observed. Involvement of the skull is particularly rare. When present, skull lesions typically appear radiographically as osteolytic without associated reactive or sclerotic changes [10-12].

Finally, an ethnic predisposition among individuals of Black or African ancestry has been recognized for several decades, with studies reporting a prevalence approximately three times higher in this population compared to other groups [1,13,14].

## Case presentation

**Figure 1:**
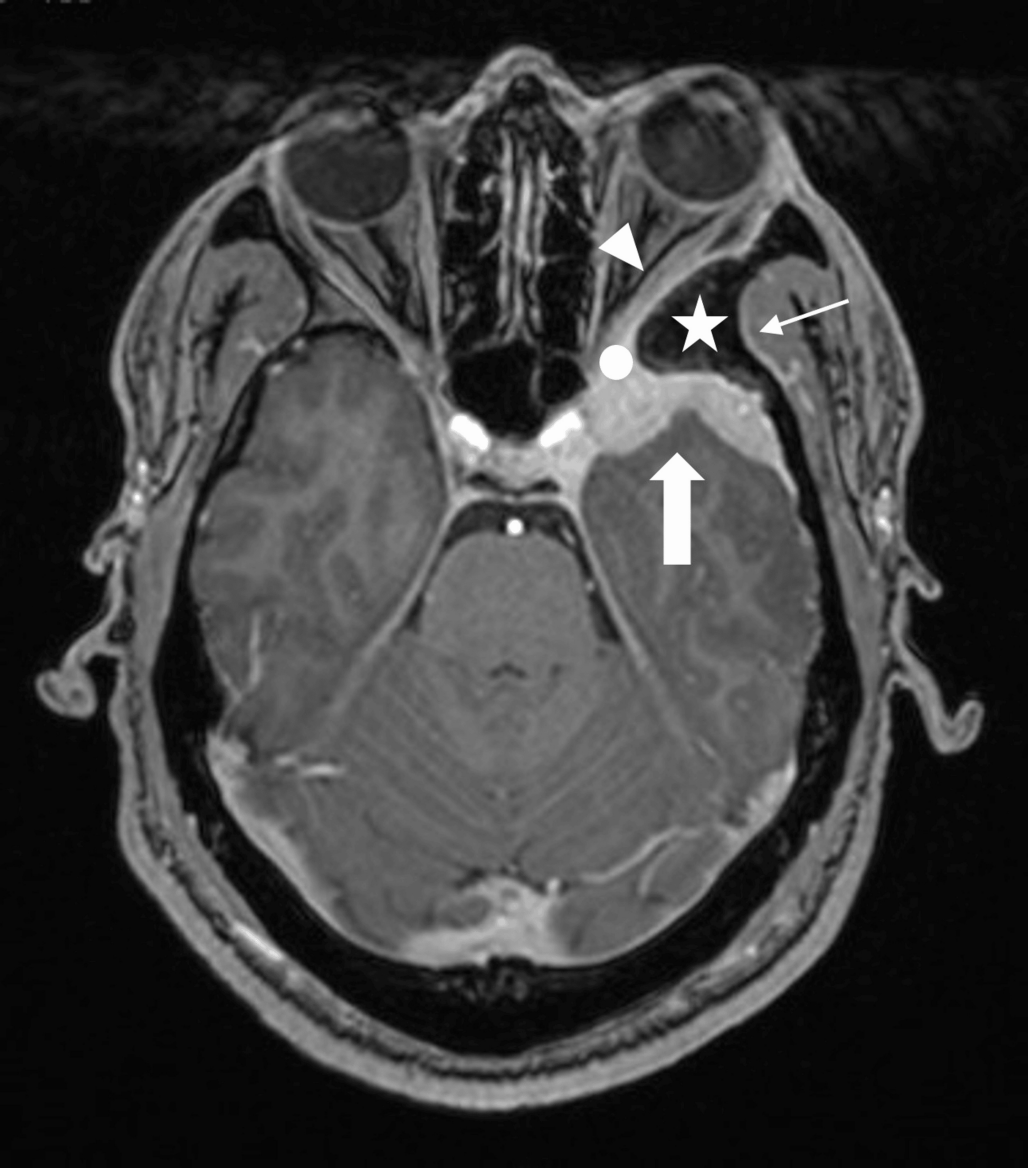
Preoperative axial T1-weighted brain MRI with gadolinium contrast. Enhancing lesion centered on the left greater wing of the sphenoid bone (white star), with associated meningeal thickening at the temporal apex (thick arrow), involvement of the orbital canal (white circle), extension into the orbit (arrowhead), and anterior portion of the cavernous sinus. Periosteal enhancement is also noted in the left temporal fossa (thin arrow).

**Figure 2:**
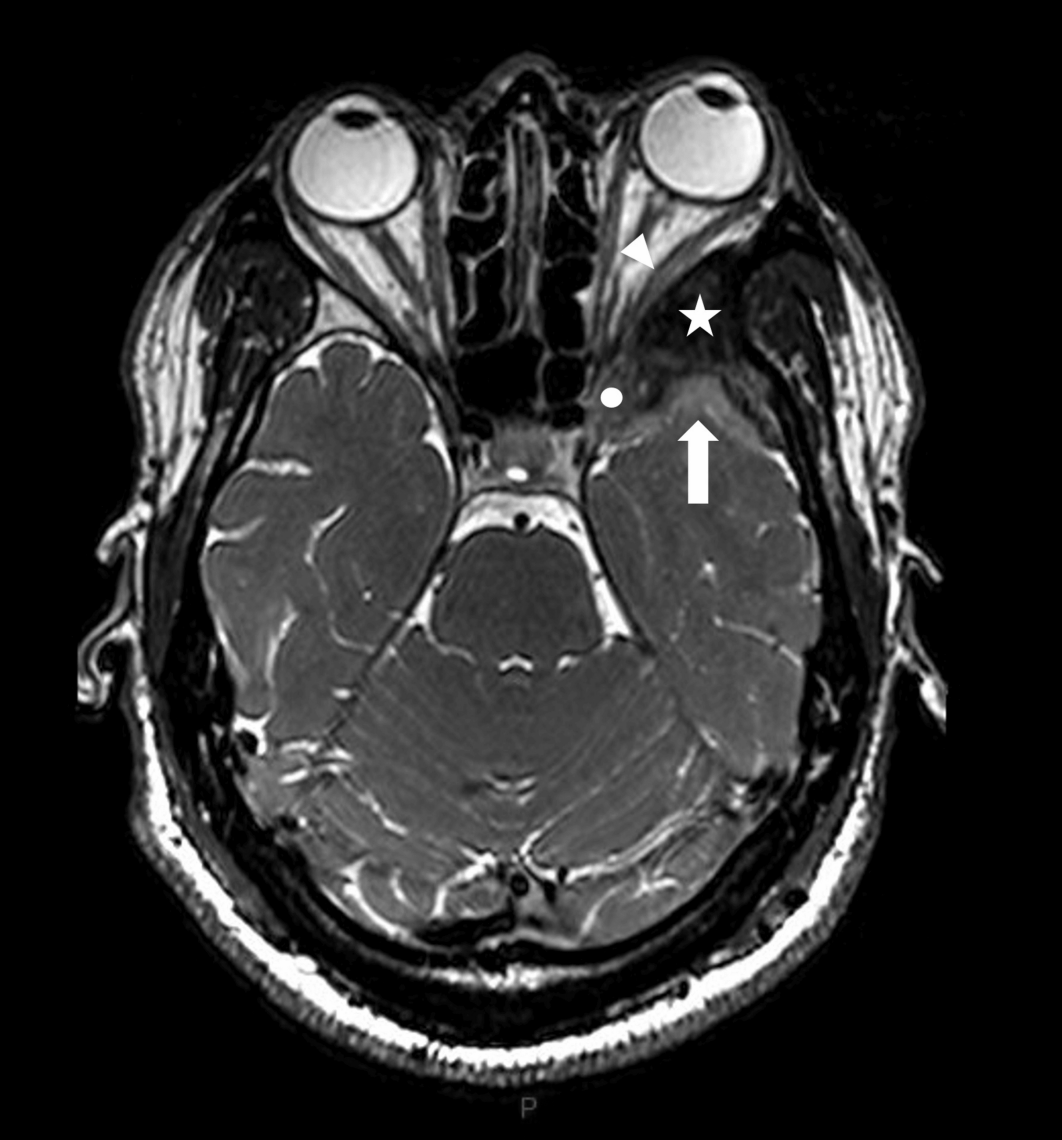
Preoperative axial T2-weighted brain MRI. The lesion is centered on the left greater wing of the sphenoid bone (white star), with associated meningeal thickening at the temporal apex (thick arrow), involvement of the orbital canal (white circle), extension into the orbit (arrowhead), and the anterior portion of the cavernous sinus.

A 34-year-old man from West Africa presented with a three-year history of retro-orbital headaches associated with mild left-sided exophthalmos. An initial brain MRI performed at another institution revealed a spheno-orbital extra-axial mass with an enhancing temporopolar and intra-orbital component, without perilesional T2-hypersignal. The primary radiological diagnosis was a spheno-orbital meningioma. The patient exhibited mild clinical signs at presentation; clinical and radiological follow-up was initially recommended. Subsequently, the patient reported the onset of blurred vision in the left eye and mild horizontal diplopia, despite a reassuring ophthalmologic examination that revealed only exophthalmos. A new evaluation was conducted at our institution. The MRI revealed a slight increase in the size of the enhancing lesion and grade 1 exophthalmos (Figures 1, 2).

**Figure 3:**
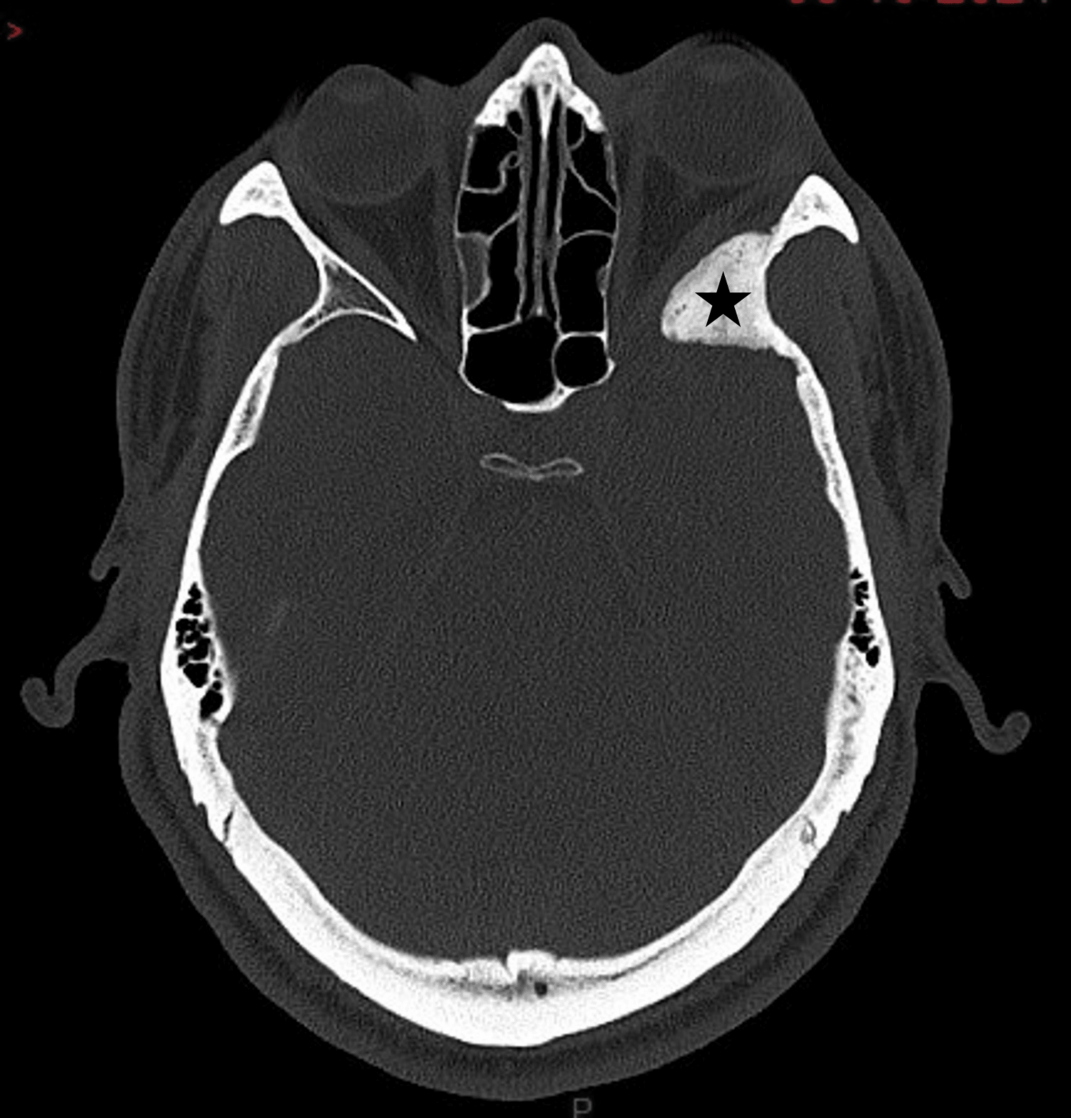
Preoperative axial non-contrast brain CT in bone window. Thickening and hyperostosis of the left greater wing of the sphenoid (black star). The margins of the hyperostosis are smooth.

Due to the presence of hyperostosis on MRI, a CT scan of the brain was performed, confirming hyperostotic changes involving the lesser and greater wings of the sphenoid, extending to the skull base, the left anterior clinoid process, and the orbit (Figure 3).

**Figure 4:**
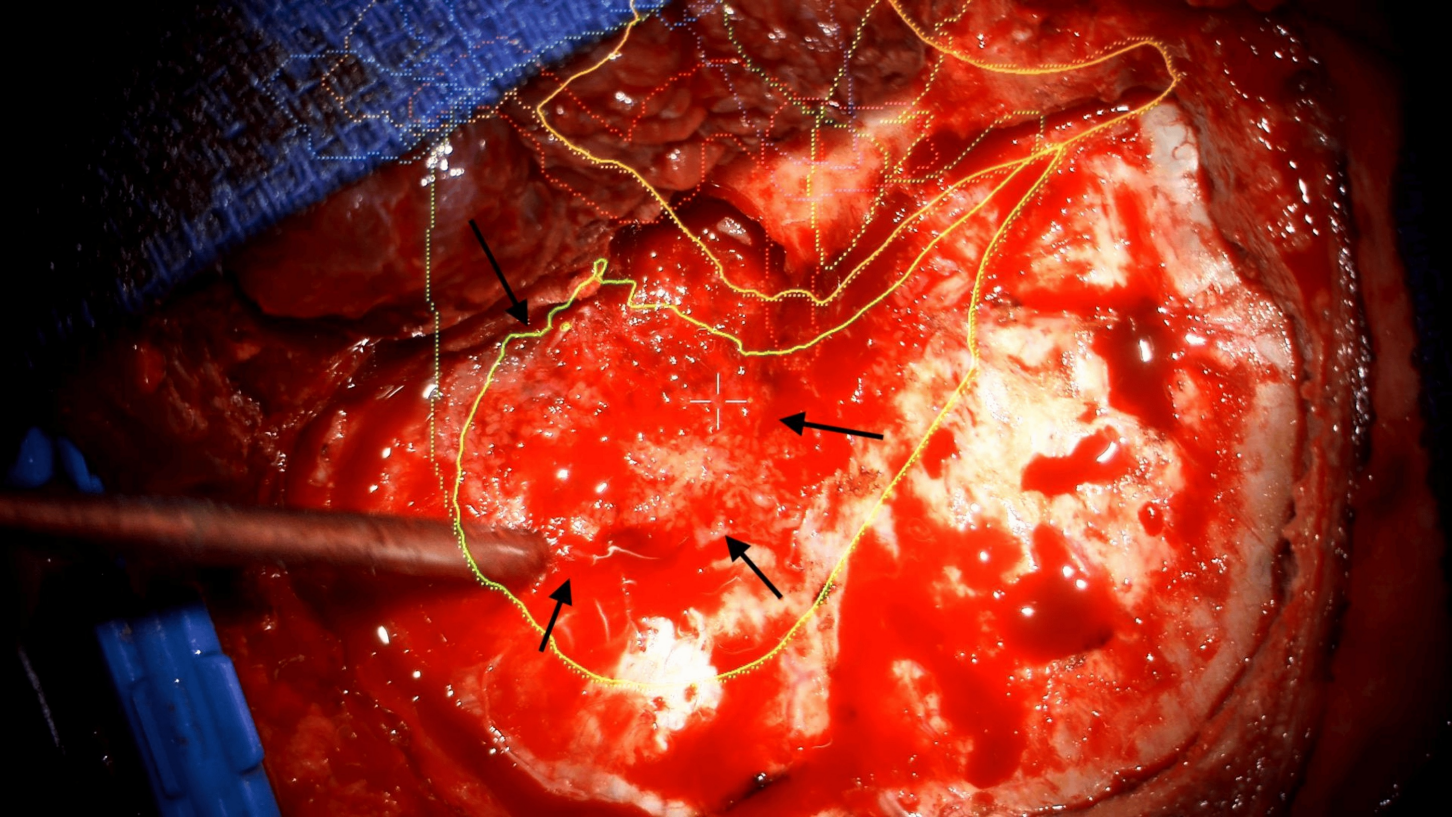
Intraoperative view showing hyperostosis of the sphenoid bone. The hyperostotic bone is delineated by black arrows.

**Figure 5:**
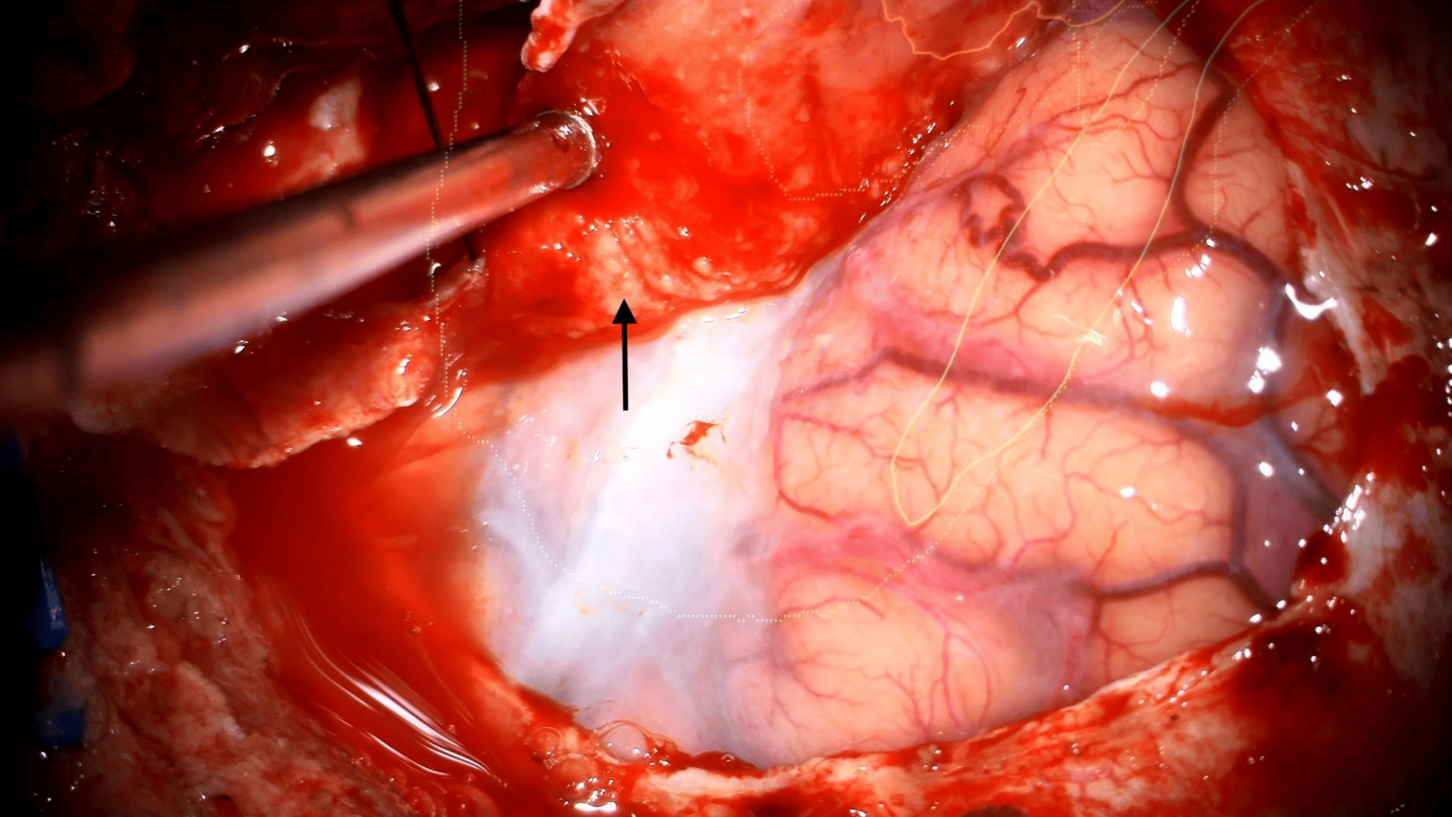
Intraoperative view after opening of the dura showing the extra-axial mass compatible with a meningioma. Extra-axial mass (black arrow).

Given the tumor progression in a more symptomatic patient, surgical resection was undertaken. The procedure involved removal of both intracranial and intra-orbital tumor components, along with drilling of all visibly affected bone (Figure 4). A small tumor portion infiltrating the superior orbital fissure was left unresected. Intraoperatively, the lesion appeared consistent with a meningioma (tan, firm, fibrous mass) (Figure 5), although there was no identifiable arachnoid plane intracranially, and marked adherence to the periorbita was noted. Bony defects were reconstructed using a mesh plate. Postoperative recovery was uneventful.

**Figure 6:**
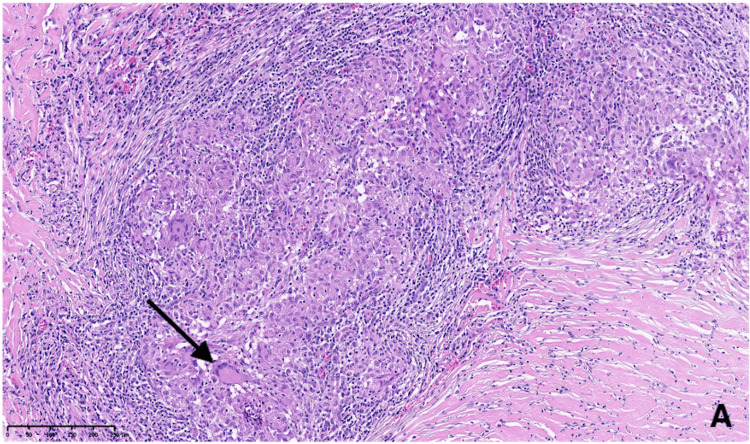
Anatomopathological analysis A. Hematoxylin and eosin (H&E)-stained sections showing epithelioid granulomas composed of epithelioid cells, Langerhans-type giant cells (black arrow).

**Figure 7:**
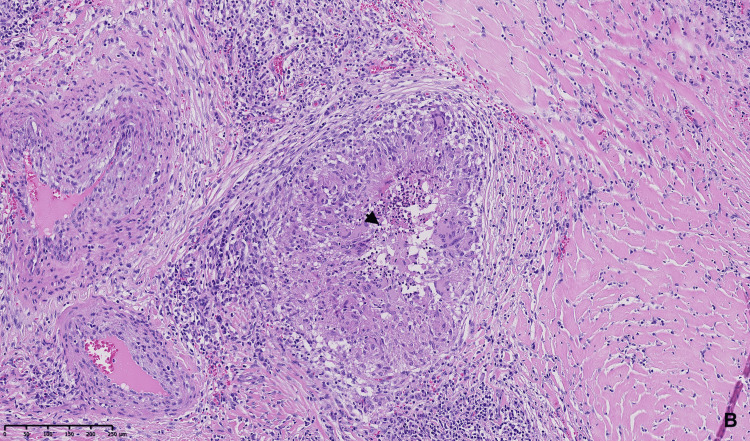
Anatomopathological analysis B. Hematoxylin and eosin (H&E) stained sections showing central necrosis (black arrowhead).

**Figure 8:**
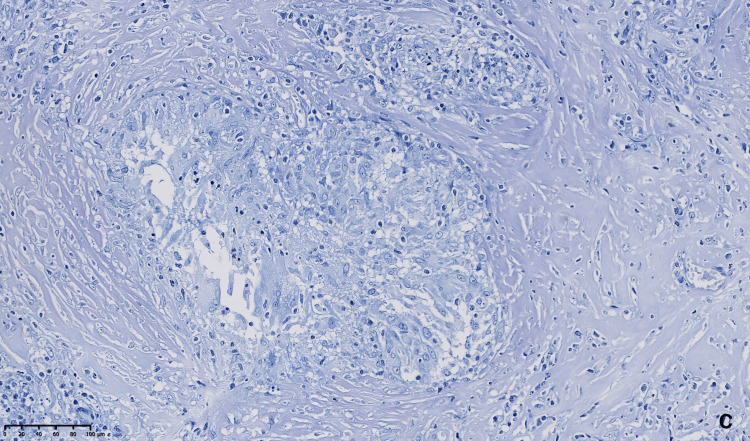
Anatomopathological analysis C. Ziehl–Neelsen staining was negative.

Histopathological analysis revealed an epithelioid granulomatous inflammatory process, consistent with sarcoidosis, with no features suggestive of meningioma (Figures 6-8).

**Figure 9:**
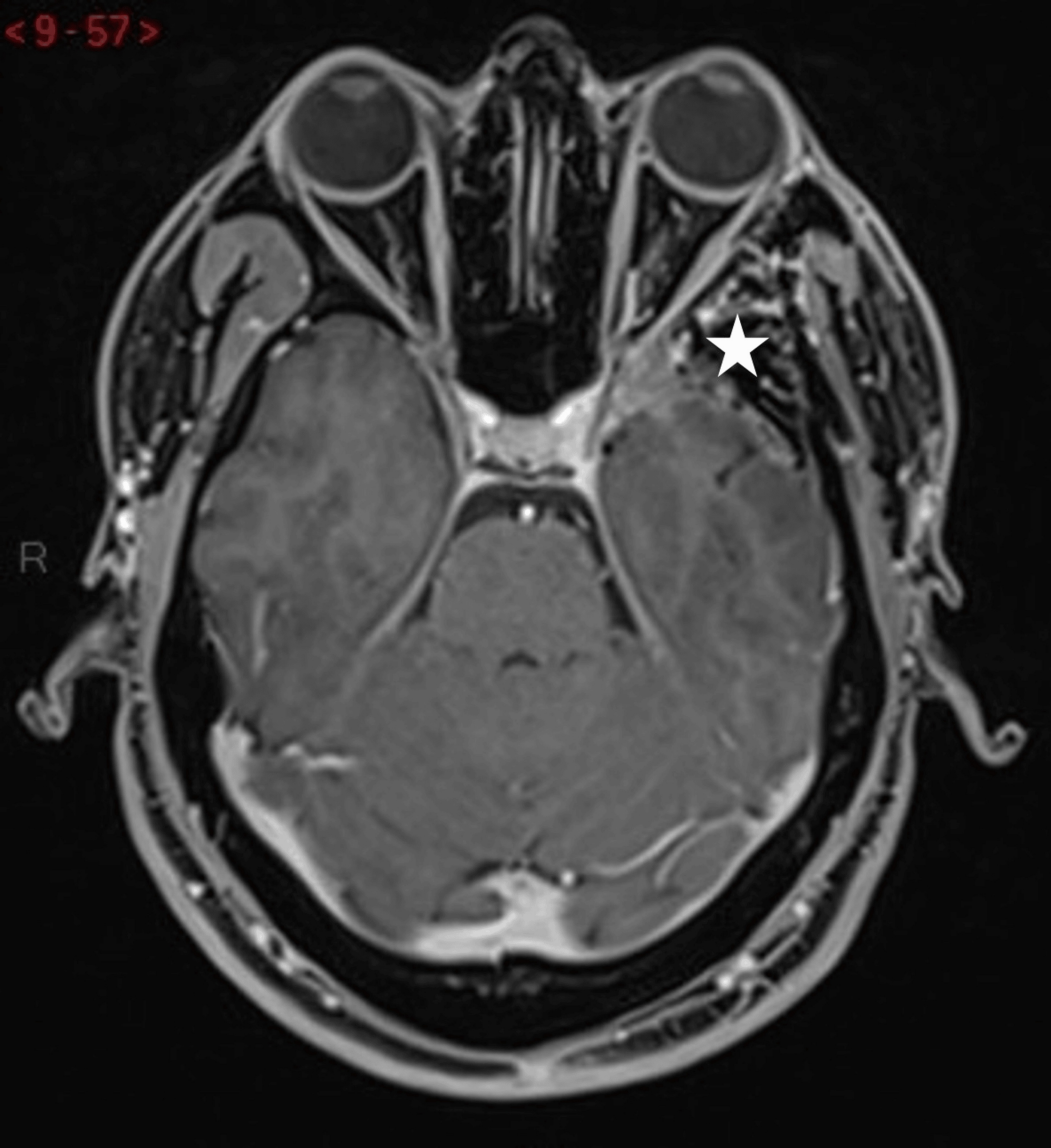
Postoperative axial T1-weighted brain MRI with gadolinium showing complete resection of the different components of the lesion (white star). Complete resection of the different components of the lesion (white star).

**Figure 10:**
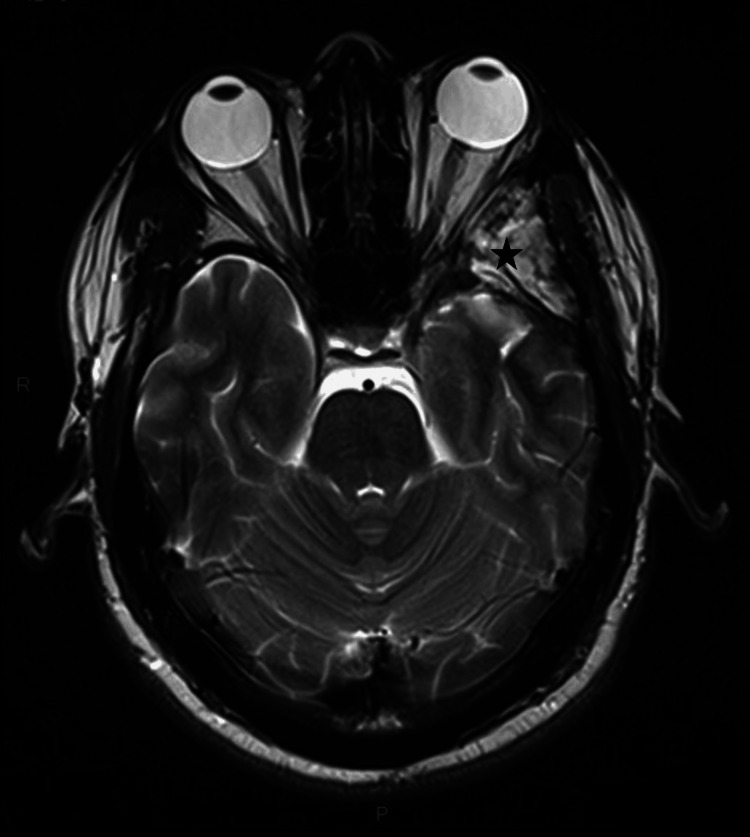
Postoperative axial T2-weighted brain MRI. Complete resection of the different components of the lesion (black star).

The patient was treated with corticosteroids, initially administered intravenously and then transitioned to oral therapy, along with oral methotrexate. Postoperative MRI imaging performed at two months showed no residual tumor and no complications (Figures 9, 10).

## Discussion

We report a rare case of neurosarcoidosis presenting as a spheno-orbital mass with associated hyperostosis of the sphenoid wing. Although uncommon, dural involvement occurs in approximately 30-35% of neurosarcoidosis cases and can radiologically manifest as intracranial extra-axial lesions, often mimicking tumors such as meningiomas [3-5,7,8].

A wide range of other pathologies may also involve the dura, including benign and malignant neoplasms, such as lymphoma, hemangiopericytoma, metastases, solitary fibrous tumors, melanocytic neoplasms, Epstein-Barr virus-associated smooth muscle tumors, and Rosai-Dorfman disease, as well as infectious and inflammatory entities like tuberculosis and sarcoidosis. These lesions frequently exhibit imaging characteristics similar to those of meningiomas, which can result in diagnostic confusion. However, many typically lack the hyperostosis often seen with meningiomas, features that may aid in radiological differentiation [15]. The distinguishing MRI characteristics of meningioma and neurosarcoidosis with dural involvement are summarized in Table 1 [4,15].

**Table 1:** MRI features differentiating meningioma from neurosarcoidosis with dural involvement. Modified from and with permission of Valappil et al. [4].

Lesion	T1-Weighted MRI	T2-Weighted MRI	T1-Weighted MRI + Gadolinium	Additional Characteristics
Meningioma	Isointense; occasionally hypointense	Isointense; occasionally hyperintense	Typically homogeneous and intense	May show calcifications, adjacent skull hyperostosis, and dural tail
Neurosarcoidosis	Hypointense or isointense	Hypointense or isointense	Typically intense, but variable	No bone involvement; may lack dural tail

The presence of sphenoid wing hyperostosis in our patient was a key factor that misled our initial diagnosis toward a meningioma. The reported incidence of hyperostosis in meningiomas varies significantly across the literature, ranging from 5% to 50%, depending on the tumor's subtype and anatomical location [16,17]. Unlike conventional meningiomas, spheno-orbital meningiomas are characteristically associated with hyperostosis, which often contributes to clinical manifestations such as proptosis [18,19].

To date, the mechanisms by which meningiomas influence the metabolic pathways underlying osteolysis and osteogenesis remain poorly understood. In meningiomas, hyperostosis is typically defined as hyperplasia of the bone adjacent to the tumor. However, some authors describe a continuum of osseous involvement, ranging from the absence of bone invasion to the presence of both hyperostosis and direct tumor infiltration of the bone. Proposed mechanisms include direct invasion, which alters osteoblast and osteoclast activity, or a humoral pathway involving the overexpression of osteogenic factors. Hyperostosis does not appear to correlate with the CNS WHO grade or the histological subtype of the meningioma [16,17].

While hyperostosis is commonly associated with meningiomas, skull involvement in osseous sarcoidosis is exceedingly rare but has been reported. Moreover, when present, these lesions most commonly exhibit an osteolytic pattern [10-12].

In our case, chronic inflammation, potentially in combination with angiogenesis, may represent a plausible mechanism underlying the observed hyperostosis. Angiogenesis is known to facilitate the recruitment and activation of osteoblasts by ensuring an adequate blood supply, thereby promoting bone formation. This vascular remodeling, in the setting of persistent inflammation, could contribute to abnormal bone proliferation and localized hyperostotic changes [20].

## Conclusions

Neurosarcoidosis should be included in the differential diagnosis of dural-based lesions, particularly when clinical or radiological features deviate from the typical presentation of meningioma. While hyperostosis is often considered a hallmark of spheno-orbital meningiomas, this case demonstrates that similar findings may rarely occur in sarcoidosis. Understanding the full clinical context, including demographic factors, systemic signs, and imaging variability, is essential for guiding appropriate management and avoiding misdiagnosis.
